# Significant risk of repeat adverse outcomes in recurrent gestational diabetes pregnancy: a retrospective cohort study

**DOI:** 10.1186/s40842-023-00149-2

**Published:** 2023-03-15

**Authors:** Sue Lynn Lau, Alex Chung, Joanna Kao, Susan Hendon, Wendy Hawke, Sue Mei Lau

**Affiliations:** 1 Western Sydney University, Campbelltown, NSW Australia; 2 Blacktown-Mount Druitt Hospital, Blacktown, NSW Australia; 3 The Prince of Wales Clinical School, UNSW, NSW Randwick, Australia; 4 The Royal Hospital for Women, Randwick, NSW Australia; 5 Department of Diabetes and Endocrinology, Prince of Wales Hospital, NSW Randwick, Australia

**Keywords:** Gestational diabetes mellitus, Large for gestational age, Small for gestational age, Pregnancy

## Abstract

**Background:**

The risk of adverse outcomes in recurrent GDM pregnancy has not been well documented, particularly in women who have already had an adverse outcome. The aim of this study was to compare the risk of recurrent adverse delivery outcome (ADO) or adverse neonatal outcome (ANO) between consecutive gestational diabetes (GDM) pregnancies.

**Methods:**

In this retrospective study of 424 pairs of consecutive (“index” and “subsequent”) GDM pregnancies, we compared the risk of ADO (instrumental delivery, emergency Caesarean section) and ANO (large for gestational age (LGA and small for gestational age (SGA)) in women with and without a history of adverse outcome in their index pregnancy.

**Results:**

Subsequent pregnancies had higher rates of elective Caesarean (30.4% vs 17.0%, *p* < 0.001) and lower rates of instrumental delivery (5% vs 13.9%, *p* < 0.001), emergency Caesarean (7.1% vs 16.3%, *p* < 0.001) and vaginal delivery (62.3% vs 66.3%, *p* = 0.01). Index pregnancy adverse outcome was associated with a higher risk of repeat outcome: RR 3.09 (95%CI:1.30,7.34) for instrumental delivery, RR 2.20 (95%CI:1.06,4.61) for emergency Caesarean, RR 4.55 (95%CI:3.03,6.82) for LGA, and RR 5.01 (95%CI:2.73,9.22) for SGA). The greatest risk factor for subsequent LGA (RR 3.13 (95%CI:2.20,4.47)) or SGA (RR 4.71 (95%CI:2.66,8.36)) was having that outcome in the index pregnancy.

**Conclusion:**

A history of an adverse outcome is a powerful predictor of the same outcome in the subsequent GDM pregnancy. These high-risk women may warrant more directed management over routine GDM care such as altered glucose targets or increased frequency of ultrasound assessment.

## Introduction

Gestational diabetes (GDM) is defined as glucose intolerance first diagnosed in pregnancy [1]. Risk factors include age, ethnicity, family history of diabetes and obesity. Notably, a history of prior GDM confers an estimated 30–60% risk of recurrent GDM [2–7].

GDM is associated with adverse outcomes for both mother and fetus, the rates of which have been well documented [8, 9]. However, the risk of complications in recurrent GDM has not been as clearly defined. There are currently no evidence-based guidelines for managing recurrent GDM or data on which women are at highest risk of adverse outcomes.

Only four studies have examined the risk of adverse outcomes in recurrent GDM [10–12]. One retrospective study of 389 women observed higher fasting glucose levels and pre-pregnancy BMI in the second GDM pregnancy compared to the first, with no increase in LGA or adverse neonatal outcomes [10]. Another study found similar LGA rates in first-time and recurrent GDM [12]. Both studies did not examine individual level data to determine the rate of repeat adverse outcome. In contrast, another study found a higher rate of LGA in the subsequent pregnancy versus the index (22.4% vs 13.8%) [11] and another found a decreased rate of macrosomia and increased rate of SGA in the subsequent GDM pregnancy [13].

The aims of this study were to quantitate the risk of adverse delivery outcome (ADO) and adverse neonatal outcome (ANO) in consecutive GDM pregnancies. We assessed the predictive value of adverse outcome in the index GDM pregnancy on the next GDM pregnancy, in the context of other risk factors.

## Methods

### Cohort

This is a retrospective longitudinal study of 424 GDM pregnancy-pairs, conducted in two centres: the Royal Hospital for Women (RHW), a tertiary maternity hospital in Eastern Sydney, and Blacktown-Mount Druitt Hospital (BMDH), a hospital in Western Sydney with the highest annual number of births statewide. Women who attended GDM clinics from 2003–2015 with more than one GDM pregnancy were identified. Each pregnancy-pair comprised two consecutive singleton GDM pregnancies (“index “ and “subsequent” pregnancies). In women with > 2 GDM pregnancies, each set of consecutive GDM pregnancies was considered a pregnancy-pair- e.g. in a woman with three GDM pregnancies, the first and second pregnancy and the second and third pregnancy were each considered as pregnancy-pairs.

Both centres used the Australasian Diabetes in Pregnancy Society diagnostic criteria at the time of a fasting plasma glucose ≥ 5.5 mmol/L and/or a 2-h glucose ≥ 8.0 mmol/L on the 2-h 75 g oral glucose tolerance test (GTT), which was performed in women with a 1-h plasma glucose of ≥ 7.8 mmol/L after a 50 g glucose challenge at 24–28 weeks gestation. Early screening for GDM was performed in the early second trimester in women with a history of GDM in a prior pregnancy, polycystic ovarian syndrome, BMI ≥ 35 kg/m^2^, maternal age ≥ 40 years or a first-degree relative with type 2 diabetes.

Glucose targets were ≤ 5.0 mmol/L fasting and ≤ 7.0 mmol/L two hours after a meal at the RHW, and ≤ 5.5 mmol/L and ≤ 7.0 mmol/L respectively at BMDH. Women were referred to the diabetes educator, instructed on home blood glucose monitoring and a low glycemic index diet and encouraged to do 30 min of exercise per day. They attended one- to four- weekly doctor appointments at the GDM clinic. Insulin was commenced in women who did not regularly meet their blood glucose targets. Diagnostic criteria, glucose targets and guidelines for GDM management remained consistent during the study period.

### Demographic and outcome data

Data were obtained from in-house databases, medical files and the Obstetrix Clinical Database System (http://www.meridianhi.com/index.php/obstetrix), a statewide database that accesses data from the New South Wales Perinatal Data Collection, a population-based surveillance system covering all births in the state. Maternal data collected included age at estimated date of confinement, ethnicity, height, weight at booking-in, week of booking-in, week of diagnosis of GDM, results of the GTT, requirement for and starting date of insulin and/or metformin, mode of delivery and need for instrumental delivery. Preterm birth was defined as delivery before 37 weeks gestation. Early GDM was defined as GDM diagnosed before 22 weeks gestation. Instrumental delivery or emergency Caesarean section were considered adverse delivery outcomes (ADO).

Neonatal data included gestational age at delivery, sex, birth weight, shoulder dystocia and fetal or neonatal death. Birth centiles were calculated using the Perinatal Institute’s customised centile calculator (https://www.gestation.net/birthweightcentiles/birthweightcentiles.htm) which accounts for maternal height, weight, ethnicity, parity, sex of the child and gestational age at birth, for an Australian population. LGA was defined as a birth weight centile ≥ 90%, and SGA was defined as a birth weight centile ≤ 10%. At the RHW, neonatal hypoglycemia was defined as capillary blood glucose < 2.2 mmol/L. At BMDH, neonatal hypoglycemia was recorded if this diagnosis had been entered into the Obstetrix database. The primary adverse neonatal outcomes (ANO) studied were LGA and SGA. In addition, a composite ANO was defined as the presence of at least one of the following: shoulder dystocia, perinatal death, LGA or SGA.

### Ethics

This study was approved by the South Eastern Sydney Local Health District-Northern Network and the Western Sydney Local Health District Human Research Ethics Committees.

### Statistical analysis

Statistical analysis was performed using SPSS 26.0 and SAS 9.4 software. Index and subsequent pregnancies were compared using paired t-tests, Wilcoxon signed-rank tests and McNemar’s test. Chi-squared tests were used to calculate the relative risk (RR) of adverse outcomes in subsequent pregnancies. Subsequent pregnancies with and without LGA, and with and without SGA, were compared using independent t-tests, Mann–Whitney U tests and chi-squared tests.

Binomial regression analysis was performed to estimate the RR of recurrent SGA and LGA. Potential factors identified on univariate analysis were included in the model and backward stepwise removal was performed in order to identify independent predictors of each outcome of interest and their adjusted RR.

Results are expressed as mean ± standard deviation for parametric data and median and interquartile range for non-parametric data, unless otherwise stated. Critical significance is taken at 5%.

## Results

### Maternal characteristics

424 pregnancy-pairs were analysed: 170 pairs from RHW (centre 1) and 254 pregnancy-pairs from BMDH (centre 2). There were 804 pregnancies in 380 women, with 32 women having three GDM pregnancies and six having four GDM pregnancies.

Maternal characteristics in index and subsequent GDM pregnancies are shown in Table 1. The mean age was 30.6 ± 4.9 years in the index pregnancy and 33.5 ± 4.9 years in the subsequent pregnancy. Booking-in weight and BMI were higher in subsequent pregnancies (*p* < 0.001). GDM was diagnosed three weeks earlier (*p* < 0.001). The rate of medication use (insulin, metformin or both) was higher in subsequent pregnancies (63.7% vs 54.0%, *p* < 0.001) and medication was started three weeks earlier (*p* < 0.001). There were no differences in GTT results.

**Table 1:** Characteristics of index and subsequent GDM pregnancies. Data expressed as mean (SD), median (IQR) or n (%)

	GDM pregnancy-pairs (*n* = 424)		
	Index	Subsequent	*p* value
Age (years)	30.6 (4.9)	33.5 (4.9)	< 0.001
Booking-in weight (kg)	67 (58.0,82.0)	70 (59.0,87.0)	< 0.001
Booking-in BMI (kg/m^2^)	26.2 (22.6, 31.6)	27.1 (23.4,32.5)	< 0.001
Week of booking	12 (6.0)	13 (5.7)	0.42
Ethnicity (n,%)			
-Europid	178 (42.0)		
-East Asia	89 (21.0)		
-South Asia	68 (16.0)		
-Middle East	57 (13.4)		
-Other	32 (7.6)		
Parity (n,%)			
0	238 (56.1)	-	< 0.001
1	104 (24.5)	223 (52.7)	
2	45 (10.6)	110 (26.0)	
> 2	37 (8.7)	90 (21.3)	
Week of GDM diagnosis	27 (25,29)	24 (16,27)	< 0.001
Early vs late GDM (n,%)			
-Early	41 (9.7)	180 (42.5)	< 0.001
-Late	383 (90.3)	244 (57.5)	
GTT- fasting glucose (mmol/L)	4.9 (0.9)	5.0 (1.0)	0.22
GTT- 2 h glucose (mmol/L)	9.0 (1.4)	8.9 (1.7)	0.51
Medication required (%)			
-Yes	229 (54.0)	270 (63.7)	< 0.001
-No	195 (46.0)	154 (36.3)	
Week medication started	30 (28,33)	27 (21,30)	< 0.001
Mode of delivery (%)			
-Vaginal birth	281 (66.3)	264 (62.3)	0.01
-Caesarean section	143 (33.7)	160 (37.7)	
Induction of labour (n, %)			
-Yes	200 (47.2)	143 (33.7)	< 0.001
-No	224 (52.8)	281 (66.3)	
Instrumental delivery (n, %)			
-Yes	59 (13.9)	21 (5.0)	< 0.001
-No	365 (86.1)	403 (95.0)	
Emergency Caesarean (n, %)			
-Yes	69 (16.3)	30 (7.1)	< 0.001
-No	355 (83.7)	394 (92.9)	
Elective Caesarean (n, %)			
-Yes	72 (17.0)	129 (30.4)	< 0.001
-No	352 (83.0)	295 (69.6)	
Gestation at delivery (weeks)	38.7 (1.6)	38.5 (1.4)	0.02
Delivery < 37 wks			
-Yes	30 (7.1)	40 (9.4)	0.20
-No	394 (92.9)	384 (90.6)	
Birth weight (g)	3315 (554)	3392 (587)	0.005
Birth weight centile (%)	50 (27.77)	54 (28,80)	0.23
SGA (%)			
-Yes	33 (7.8)	37 (8.7)	0.67
-No	391 (92.2)	387 (91.3)	
LGA (%)			
-Yes	71 (16.7)	67 (15.8)	0.73
-No	353 (83.3)	357 (84.2)	
Birth length (cm)	50.1 (3.0)	50.3 (2.6)	0.12
Fetal/neonatal death (n, %)			
-Yes	3 (0.7)	1 (0.2)	0.63
-No	421 (99.3)	423 (99.8)	
Dystocia (n, %)			
-Yes	17 (4.0)	8 (1.9)	0.09
-No	407 (96.0)	416 (98.1)	
Neonatal hypoglycemia (n, %)			
-Yes	57 (13.4)	69 (16.3)	0.25
-No	367 (86.6)	355 (83.7)	
Composite neonatal outcome (death/dystocia/LGA/ SGA) (n, %)			
-Yes	114 (26.9)	108 (25.5)	0.66
-No	310 (73.1)	316 (74.5)	

While there were minor differences in maternal characteristics between the centres, interpregnancy changes were generally comparable. The mean increment in age between index and subsequent pregnancies was slightly smaller in centre 1 versus centre 2 (2.6 ± 1.3 vs 3.1 ± 1.7 years, *p* = 0.001). Similarly, the mean increment in body weight at booking-in (the first antenatal visit) was smaller in centre 1 (1.8 ± 5.7 vs 3.1 ± 6.1 kg, *p* = 0.03). There were no differences in interval changes between centres for other maternal parameters including BMI at booking-in and glucose levels on the GTT.

### Adverse delivery outcomes (ADO)

Instrumental delivery (5% vs 13.9%, *p* < 0.001) and emergency Caesarean Sect. (7.1% vs 16.3%, *p* < 0.001) were decreased in the subsequent pregnancy. However, there was a higher rate of elective Caesarean Sect. (30.4% vs 17.0%, *p* < 0.001) and a lower rate of vaginal delivery (62.3% vs 66.3%, *p* = 0.01) and induction of labour (33.7% vs 47.2%, *p* < 0.001) (Table 1).

Only 2.8% of women with a Caesarean section in the index pregnancy went on to have a vaginal delivery in the next pregnancy, with 12.2% having an emergency Caesarean section and 79.7% having an elective Caesarean; this was different to women who delivered via vaginal birth in the index pregnancy, of whom 90.1% delivered via vaginal birth, 4.6% via emergency Caesarean section and 5.3% via elective Caesarean section (*p* < 0.001). Mode of delivery in those with a composite neonatal adverse outcome in the index pregnancy (vaginal delivery 55.5%, emergency Caesarean 9.2%, elective Caesarean 35.3%) was similar to those without adverse outcome (vaginal delivery 64.5%, emergency Caesarean 6.4%, elective Caesarean 29.1%) (*p* = 0.20).

### Adverse neonatal outcomes (ANO)

There were no differences in the rates of SGA, LGA, fetal/neonatal death, neonatal hypoglycemia, or the composite ANO (death/dystocia/LGA/SGA) when index and subsequent GDM pregnancies were compared as a group (Table 1). Babies from subsequent pregnancies were delivered slightly earlier (38.5 ± 1.4 vs 38.7 ± 1.6 weeks gestation, *p* = 0.02) than those from index pregnancies. While birth weight was increased in the subsequent pregnancy (3392 ± 587 g vs 3315 ± 554 g, *p* = 0.005), customised birth centiles were similar (54 (28–80) % vs 50 (27–77) %, *p* = 0.23) (Table 1).

### Risk of recurrent adverse delivery outcomes

The risk of ADO in the subsequent pregnancy was greatly increased in those women who had ADO in their index pregnancies, with a threefold risk of instrumental delivery in those women who required it in their index pregnancy, and a 2.2-fold risk of emergency Caesarean section compared to women who did not. Similarly, women who had early GDM, requirement for medication, or preterm birth in their index pregnancies were at much higher risk of developing the same outcomes in their subsequent pregnancy (Table 2).

**Table 2:** Rate and relative risk of recurrent maternal and neonatal outcomes in the subsequent GDM pregnancy

Outcome	Rate ^a^ (%) *n* = 424	Relative risk ^b^ (95% CI) in subsequent pregnancy	*p* value
Early GDM	67.5	1.65 (1.29, 2.11)	< 0.001
GDM requiring medication	87.3	2.40 (1.98, 2.91)	< 0.001
Instrumental delivery	11.9	3.09 (1.30, 7.34)	0.011
Emergency Caesarean section	13.0	2.20 (1.06, 4.61)	0.036
SGA	33.3	5.01 (2.73, 9.22)	< 0.001
LGA	45.1	4.55 (3.03, 6.82)	< 0.001
Preterm birth	33.3	4.38 (2.37, 8.07)	< 0.001
Composite neonatal outcome (death/dystocia/LGA/ SGA)	41.2	2.10 (1.53, 2.87)	< 0.001

^a^in women with this outcome in the index pregnancy

^b^compared to women without this outcome in their index pregnancy

### Risk of recurrent adverse neonatal outcomes

**Fig. 1:**
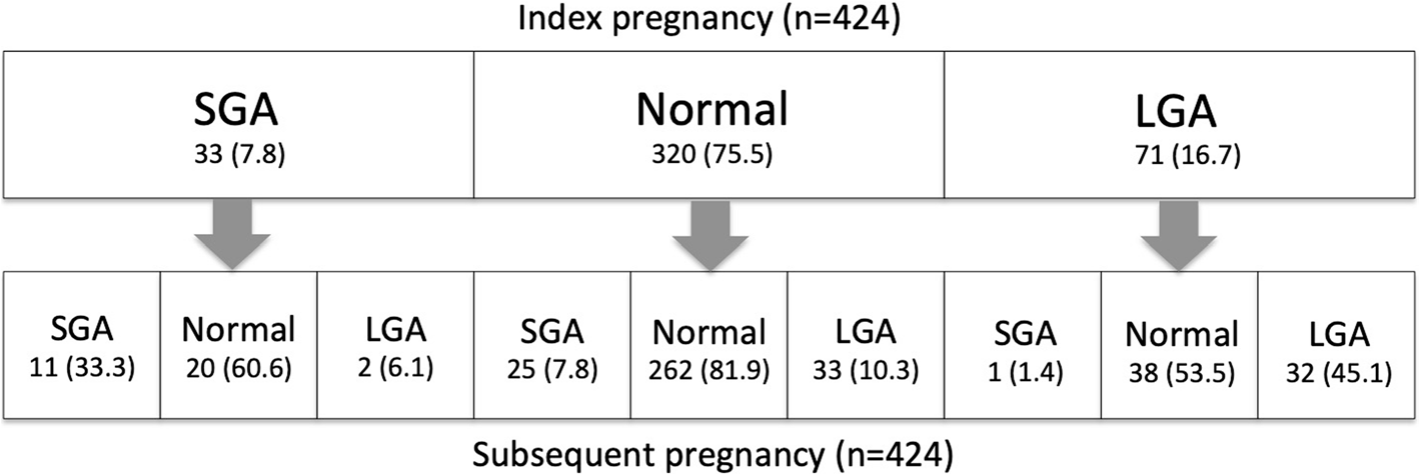
Small for gestational age (SGA) and large for gestational age (LGA) outcomes in index and subsequent pregnancies. Data expressed as n (%)

While the rate of LGA or SGA was not different in index versus subsequent pregnancies as a group, the risk of these complications in their subsequent pregnancy was greatly increased in those women who had LGA or SGA in their index pregnancy compared to those who did not (Fig. 1). For example, while the rate of LGA was similar in index and subsequent pregnancies (16.7% vs 15.8%, *p* = NS), the rate of LGA in the subsequent pregnancy was 45.1% in those women who had LGA in their index pregnancy, with a RR of 4.55 compared to women who did not. Likewise, while the rate of SGA was similar in index and subsequent pregnancies (7.8% vs 8.7%, *p* = NS), the rate of SGA in the subsequent pregnancy was 33.3% in women who had SGA in their index pregnancy, with a RR of 5.01 compared to women who did not (Fig. 1). This greatly increased risk was also the case for the composite ANO (death/dystocia/LGA/SGA) (Table 2).

Conversely, having an SGA baby in the index GDM pregnancy was associated with a below average rate of LGA (6.1%, *n* = 2/33), and having prior LGA was associated with an SGA rate of only 1.4% (*n* = 1/71) in the subsequent GDM pregnancy. Women with no LGA or SGA history had a 7.8% rate of SGA and 10.3% rate of LGA in their subsequent GDM pregnancy (Table 2).

### Factors associated with ANO in the subsequent GDM pregnancy

In women with LGA in the index pregnancy, those who went on to have another LGA pregnancy had a higher booking-in BMI in that index pregnancy (30.5 (26.6–40.7) vs 25.7 (23.1–30.6) kg/m^2^, *p* = 0.008) as well as the subsequent pregnancy (32.2 (28.3–41.9) vs 27.3 (24.0–30.4) kg/m^2^, *p* = 0.001) compared to women who did not have another LGA pregnancy. They also had a higher parity (50.0% vs 9.0% > 2, *p* = 0.02) in the index pregnancy and a higher 2 h glucose level on the GTT (10.6 ± 2.5 vs 8.8 ± 2.6 mmol/L, *p* = 0.02) compared to women who did not have a repeat LGA pregnancy. There were no differences in age or interval between pregnancies.

On univariate analysis, women with LGA in their subsequent GDM pregnancy were slightly younger with higher parity compared to those without LGA. They had a 17.5 kg greater median booking-in weight (84.5 (69.0–105.0) vs 67.0 (58.0–82.0) kg, *p* < 0.001), higher booking-in BMI (31.3 (26.9–37.4)) vs 26.5 (23.1–32.0) kg/m^2^, *p* < 0.001) and a 2.5 kg greater interpregnancy weight gain than women without LGA (4.7 ± 8.4 vs 2.2 ± 5.4 kg, *p* = 0.002), despite a similar interpregnancy interval. They had a higher fasting and two-hour glucose on the diagnostic GTT. 47.8% had LGA in their index pregnancy, whereas only 10.9% of women without LGA in the subsequent pregnancy had LGA in the index pregnancy (*p* < 0.001) (Table 3).

**Table 3:** Characteristics of subsequent GDM pregnancies with and without LGA and SGA. Data expressed as mean (SD), median (IQR) or n (%)

	No LGA (*n* = 67)	LGA (*n* = 357)	*p* value	No SGA (*n* = 387)	SGA (*n* = 387)	*p* value
Age (years)	33.7 (4.7)	32.4 (5.6)	0.02	33.5 (5.0)	33.6 (4.0)	0.94
Interval between pregnancies (years)	3.0 (1.5)	2.7 (1.7)	0.28	2.8 (1.5)	3.9 (2.1)	< 0.001
Booking-in weight (kg)	67.0 (58.0,82.0)	84.5 (69,105)	< 0.001	71.0 (59.3,88.0)	65.0 (56.0,77.5)	0.07
Weight change between pregnancies (kg)	2.2 (5.4)	4.7 (8.4)	0.002	2.6 (6.1)	2.4 (5.5)	0.87
Booking-in BMI (kg/m^2^)	26.5 (23.1,32.0)	31.3 (26.9, 37.4)	< 0.001	27.1 (23,4,32.8)	23.2 (25.8,31.6)	0.46
Ethnicity (n,%)			0.08			0.11
-Europid	38.4	50.7		41.6	27.0	
-Non-Europid	61.6	49.3		58.4	73.0	
Parity (n,%)			0.002			0.87
1	55.9	35.8		52.3	56.8	
2	25.6	28.4		26.2	24.3	
> 2	18.9	35.8		21.5	18.9	
Week of GDM diagnosis	24.0 (17.0,27.0)	23.5 (14.8,28.0)	0.89	24.0 (16.0,27.0)	20.0 (15.5,26.0)	0.10
Early vs late GDM (n,%)			0.42			0.22
-Early	42.7	48.5		42.6	54.1	
-Late	57.3	51.5		57.4	45.9	
GTT- fasting glucose (mmol/L)	4.9 (0.8)	5.6 (1.5)	< 0.001	5.0 (1.0)	4.7 (0.7)	0.27
GTT- 2 h glucose (mmol/L)	8.8 (1.6)	9.7 (2.2)	0.002	9.0 (1.7)	8.8 (1.5)	0.71
Medication required (%)			0.17			0.72
-Yes	62.5	71.6		63.3	67.6	
-No	37.5	28.4		36.4	32.4	
Same outcome in previous GDM preg			< 0.001			< 0.001
-Yes	10.9	47.8		5.7	70.3	
-No	89.1	52.2		94.3	29.7	

In women with SGA in the index pregnancy, those who went on to have another SGA pregnancy had a longer interval between pregnancies (4.8 ± 2.5 vs 2.8 ± 1.3 years, *p* = 0.04) compared to those who did not have another SGA pregnancy. There were no differences in age, parity, booking-in BMI or GTT results.

On univariate analysis, women with SGA in their subsequent GDM pregnancy had a longer interpregnancy interval (3.9 ± 2.1 vs 2.8 ± 1.5 years, *p* < 0.001) compared to women without SGA. 70.3% had SGA in their index pregnancy, versus 5.7% of women without SGA in their subsequent pregnancy (*p* < 0.001). There was a trend to lower booking-in weight (65.0 (56.0–77.5) vs 71.0 (59.3–88.0) kg, *p* = 0.07) but no differences in booking-in BMI or interpregnancy weight change (Table 3).

Based on results of univariate analysis, potential predictors of LGA in the subsequent pregnancy were included in a binomial regression model (prior LGA, BMI at booking-in, interpregnancy weight gain, and fasting glucose at diagnostic OGTT). After backward stepwise removal, LGA in the index pregnancy remained the strongest predictor of subsequent LGA, with a RR of 3.13 (95%CI:2.20, 4.47, *p* < 0.001) compared to women without prior LGA. Booking-in BMI showed a modest association with LGA outcome- RR 1.04 (95%CI:1.02, 1.07, *p* < 0.001).

For the outcome of SGA in the subsequent pregnancy, prior SGA, interpregnancy interval and booking-in weight were included in the model. After adjustment, the RR of SGA in women with SGA in the index pregnancy was 4.71 (95%CI:2.66, 8.36, *p* < 0.001). For every one-year increase in the interpregnancy interval, the RR of SGA was 1.51 (95%CI:1.19, 1.91, *p* < 0.001.

## Discussion

In this study of 424 pairs of consecutive GDM pregnancies, an ADO or ANO in the index GDM pregnancy conveyed a greatly increased risk of the same outcome in the subsequent GDM pregnancy. While these risks have been described in the general antenatal population, they have not been previously quantitated in GDM.

Compared to index GDM pregnancies, the rates of instrumental delivery and emergency Caesarean section were more than halved in subsequent pregnancies, with correspondingly increased rates of elective Caesarean section, lower rates of vaginal delivery and induction of labour. This could be explained by a greater consideration of elective Caesarean in women who had a Caesarean section in the index pregnancy, with only 2.8% of these women delivering vaginally in the subsequent pregnancy, and nearly 80% delivering via elective Caesarean. At our centre, all women with a prior Caesarean section are counseled about the small but significant risk of uterine rupture in a subsequent labour and the majority choose to have an elective Caesarean.

While ADOs were improved in subsequent pregnancies, the risk of having an ADO was still far greater in women with a history of the same ADO in the index pregnancy, with a RR of 3.09 for instrumental delivery and 2.20 for emergency Caesarean. These risks may justify a lower threshold for elective Caesarean in women with recurrent GDM and history of instrumental delivery or emergency Caesarean.

While delivery outcomes were improved in subsequent GDM pregnancies, ANO rates were unchanged in index versus subsequent pregnancies, with LGA rates of ~ 16%, SGA rates of ~ 8% and overall composite ANO rates of ~ 26%. Given that women with subsequent GDM pregnancies were older, had a higher BMI and were more likely to require medication, it could be hypothesised that they should have had a higher rate of adverse outcomes, which was not the case. One explanation could be that women were diagnosed earlier due to earlier screening which may have affected ANO rates. 

Four retrospective studies have examined the comparative rates of LGA in first and second GDM pregnancies [10–13], although none have analyzed detailed individual-level data across consecutive pregnancies. Two studies [10, 12] also found that LGA and SGA rates were not significantly different between pregnancies with first time recurrent GDM. In a study of GDM pregnancy-pairs, [11]. LGA rate increased in the second GDM pregnancy (22.4% vs 13.8%, *p* < 0.05). The risk of recurrent LGA was 55.7%, comparable to our rate of 45.1%. It did not examine other ANOs such as SGA and fetal or neonatal death, and clinical information such as timing of diagnosis of GDM, results of the GTT, maternal BMI and interpregnancy interval were not included in the analyses. Thus, they were not able to evaluate for effects of other factors associated with increased risk of recurrent LGA. A fourth study [13] found a decreased rate of SGA in 56 Chinese women wtih recurrent versus index GDM pregnancies. However, this study was confined to diet-controlled GDM.

Our study lends a new perspective by tracking the incidence of ANOs in individual women over consecutive GDM pregnancies. The RR of repeat outcome for women with LGA, SGA or any ANO in their GDM pregnancy was 4.5 fold, 5.0 and 2.1 respectively. Put another way, nearly half of women with LGA, 70% of women with SGA and 44% of women with the composite ANO in their subsequent pregnancy had the same outcome in their index pregnancy. Thus while ANO rates were similar in index and subsequent pregnancies as a group, a substantial proportion of adverse outcomes were occurring in the same women. Of additional interest is the very low risk of SGA in women with prior LGA, and the low risk of LGA in women with prior SGA.

Multivariate analysis of ANO in the subsequent pregnancy showed that having the same outcome in the index pregnancy was by far the strongest risk factor, with a 3.1-fold risk of LGA and 4.7-fold risk of SGA. In LGA, this risk far outweighed that of maternal BMI, a well-established risk factor for LGA [14, 15]. In SGA, this risk far outweighed that associated with increasing interpregnancy interval.

The risks of recurrent LGA or SGA have not been previously described in GDM. Our calculated risks are similar in magnitude to the five-fold risk observed in general obstetric cohorts for LGA [16, 17] as well as for SGA [18, 19]. Moreover, severity of GDM based on the GTT results, need for medication and timing of diagnosis were not associated with subsequent pregnancy outcomes. While on the surface these data may suggest that GDM does not impact greatly on risk of recurrent ANOs, we were not able to include the modifiable factors of gestational weight gain and a measure of glycaemia such as HbA1c in our model, and cannot discount the importance of weight and glycemic management.

Limitations of this study include unavailable data on maternal smoking status, gestational weight gain, hypertensive disorders as well as weight gain and glycemic control during pregnancy. There were slight differences between centres in glucose targets and population demographics although reassuringly, interpregnancy changes did not differ between centres. We relied on medication requirement as a surrogate marker of glycaemia. It is possible some of the participants may have had undiagnosed type 2 diabetes as the ADIPS criteria for GDM does not specifically exclude women who may have had undiagnosed type 2 diabetes prior to pregnancy.

Strengths of this study included the inclusion of consecutive GDM pregnancy-pairs and the analysis of longitudinal data in individual patients that allowed us to assess risk of adverse outcomes in the context of previous complications. We were able to adjust for relevant clinical covariates including interpregnancy duration and interpregnancy weight gain, both pertinent to recurrent GDM. The definitions of LGA and SGA were based on customised centiles for an Australian population.

According to current standards, diagnostic criteria, glucose targets and weight gain targets are applicable to all women with GDM, irrespective of their history of ADO/ANO. There are currently no evidence-based guidelines for managing GDM. Our data support a more individualised management of GDM in those with previous ADO and ANO.

## Conclusions

While rates of ANO were similar in index and subsequent GDM pregnancies, the risk was greatly increased in women who had ANO in the index pregnancy. This is despite decreased rates of ADO. Our study identifies a group of women with recurrent GDM and previous LGA who may stand to gain the most from intensive management of their glucose levels and weight. This may include tighter glucose targets and/or more frequent ultrasound assessment. Our study also identifies a group of women with recurrent GDM and previous SGA in whom intensive or early therapy might potentially be unwarranted, given the high risk of recurrent SGA and the low risk of LGA.

## Data Availability

The datasets used and/or analysed during the current study are available from the corresponding author on reasonable request.
